# Electrohydrodynamics Analysis of Dielectric 2D Nanofluids

**DOI:** 10.3390/nano12091489

**Published:** 2022-04-27

**Authors:** Mrutyunjay Maharana, Niharika Baruah, Sisir Kumar Nayak, Niranjan Sahoo, Kai Wu, Lalit Goswami

**Affiliations:** 1 School of Electrical Engineering, SKLIPE, Xi’an Jiaotong University, Xi’an 710049, China; wukai@xjtu.edu.cn; 2 Departement of Mechanical Engineering, DRIEMS Cuttack, Cuttack 754022, India; 3 Indian Institute of Technology, Guwahati 781039, India; b.niharika@iitg.ac.in (N.B.); sknayak@iitg.ac.in (S.K.N.); shock@iitg.ac.in (N.S.); 4 Department of Chemical Engineering, Chungbuk National University, Cheongju-si 28644, Korea; lalitgoswami660323@gmail.com

**Keywords:** nanodielectrics, electrophoresis, breakdown, voltage, charge dynamics, 2D nanomaterials, thermal conductivity

## Abstract

The purpose of this present study is to prepare a stable mineral-oil (MO)-based nanofluid (NF) for usage as a coolant in a transformer. Nanoparticles (NPs) such as hexagonal boron nitride (h-BN) and titanium oxide (TiO_2_) have superior thermal and electrical characteristics. Their dispersion into MO is likely to elevate the electrothermal properties of NFs. Therefore, different batches of NFs are prepared by uniformly dispersing the insulating h-BN and semiconducting TiO_2_ NP of different concentrations in MO. Bulk h-BN NP of size 1μm is exfoliated into 2D nanosheets of size 150–200 nm, subsequently enhancing the surface area of exfoliated h-BN (Eh-BN). However, from the zeta-potential analysis, NP concentration of 0.01 and 0.1 wt.% are chosen for further study. The thermal conductivity and ACBDV studies of the prepared NF are performed to investigate the cooling and insulation characteristics. The charging-dynamics study verifies the enhancement in ACBDV of the Eh-BN NF. Weibull statistical analysis is carried out to obtain the maximum probability of ACBDV failure, and it is observed that 0.01 wt.% based NF has superior cooling and insulation properties than MO and remaining batches of NFs.

## 1. Introduction

The transformer is a critical link in a power system, and its reliability is crucial to the continuity of the power supply. It is subjected to multiple stresses such as electrical, mechanical, chemical, and thermal, simultaneously. The insulation system of the transformer mostly comprises oil and cellulosic kraft paper, which is the weakest element. While oil can be replaced without opening up the transformer, any damage to cellulose requires the transformer to be opened up for repair or replacement, which is a long, drawn-out process. The transformer shutdown causes significant disruption to the power supply and should be minimized. Hence, efforts are being taken at both the design and operation stage to arrest the aging rate of cellulose. 

The cellulose present inside a transformer ages fast in the presence of heat and moisture, as it is subjected to high temperature, being next to the heat-generating conductor. The oil used as coolant and insulant in the transformer plays a crucial role. The higher the thermal conductivity of oil, the lower the temperature rise on the cellulose will be. Cellulose is hygroscopic and holds much more water than oil. If the moisture equilibrium between oil and cellulose can be altered such that oil can take away more water from the cellulose when hot, then cellulose aging will be lower [1].

In the recent past, dielectric nanofluids (NFs) have been developed by dispersing nanoparticles (NPs) in the mineral oil (MO), which exhibits superior insulating and cooling properties [2,3]. Using various NFs, the thermal properties of base fluids could be improved by choosing suitable volume fractions of desired NPs [4,5,6,7,8]. In addition to thermal conductivity and moisture-holding ability, another desirable feature would be a higher dielectric strength [9,10,11,12]. With normal MO having a dielectric constant almost a third of cellulose, oil takes more electrical stress than cellulose. With oil having lower dielectric strength, this leads to a higher oil duct and size and hence the cost of the transformer. Therefore, any increase in the dielectric constant and/or dielectric strength of oil would help to reduce the size of the transformer [13,14]. To provide efficient cooling in a power or distribution transformer, a novel NP has to disperse in the heat-transfer fluid to exhibit very high thermal conductivity in combination with excellent electrical properties [15,16]. Among the various types of NPs, it is observed that the titanium oxide (TiO_2_) enhances the AC breakdown voltage (ACBDV) of the MO, whereas early sedimentation and the hydrophilic surface of TiO_2_ NP restricts its application in the transformer. It is known from the literature that due to the strong Van der Waals force of attraction between the bulk h-BN powders, early sedimentation is experienced in the NF [17,18,19].

Considering parameters such as ACBDV, thermal conductivity, and stability of the NFs, an exfoliation process of hexagonal boron nitride (h-BN) is carried out. This process converts 1 µm size nanopowder to 2D nanosheets of 150–300 nm [19]. It is observed that exfoliated h-BN (Eh-BN) is a new batch of insulating NP, which can upsurge the thermal conductivity of the NF. The high value of thermal conductivity and the well-suited particle surface for exfoliation [20,21] are used for the application in heat transfer. By seeking the heat-transfer and insulation properties of Eh-BN 2D nanofiller, 0.01–0.1 wt.% of the particle are dispersed into MO, and necessary electrical and thermophysical properties of Eh-BN/MO-NF are noted.

The transformer oil has two major functionalities, namely insulation and heat-transfer characteristics, which need to be optimized for a smooth and efficient transformer operation. An effective liquid dielectric is a must for an oil-filled transformer whose key role is maintaining the sound health of the transformer. Therefore, the authors studied an NF-based transformer oil to meet both criteria to be fulfilled for insulation and cooling simultaneously. The authors have also addressed the theoretical reason behind the enhancement of the heat-transfer and insulation characteristics of the NF. Thermal performance and charge-dynamics studies are performed to justify that the proposed NF has the superior potential to be an alternative dielectric fluid for transformers compared to conventional mineral oil.

## 2. Experimental Methods

### 2.1. Characterization of NPs and MO

NF was processed by using MO as base fluid, and the significant measured properties of the fresh MO are given in Table 1. Nanofillers such as h-BN and TiO_2_ powder of primary particle size of 1 μm and 21 nm and purity 98% and 99.5% were procured from Sigma-Aldrich (St. Louis, MO, USA). The properties of the nanofillers are shown in Table 2.

The primary particle dimension of the h-BN is bulk, and forms large spherical agglomerates. Therefore, to obtain the dispersion stability, bulk h-BN NP underwent a surface-treatment process. The surface of bulk h-BN was modified by the exfoliation process to obtain the 2D nanosheets [19].

### 2.2. Preparation of NFs

**Figure 1:**
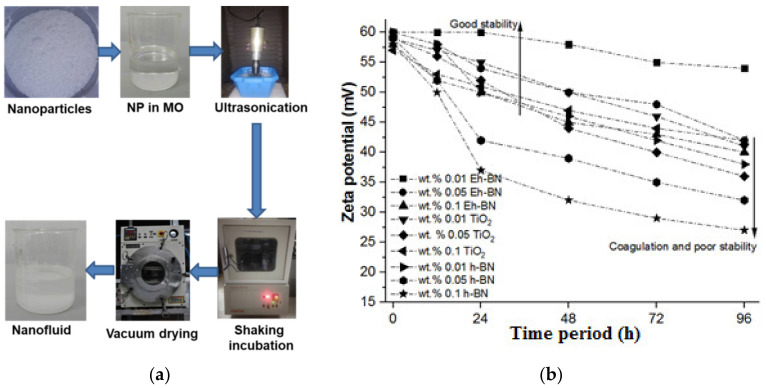
(**a**) Preparation of nanofluids; (**b**) stability of NFs at different concentrations of nanofiller.

A two-step method was followed to prepare a stable MO-based NF, as shown in Figure 1a. Nanofillers of a specific concentration were measured and mixed with MO. The sonication technique was followed to obtain the uniform scattering of NP in the based fluid. The semi-prepared solution was kept in a bath sonicator for an hour to weaken the attractive force of Van der Waals among the NPs. Since the energy required for the dispersion of the NP in the MO is not sufficient, it underwent further treatment. Thereafter, the semi-dispersed NF was undertaken for probe-sonication for an hour at room temperature. The temperature of the NF during the sonication was maintained by keeping the ice cubes around the beaker. The uniformly dispersed NF contained moisture, for which further physicochemical and electrical analysis could not be performed. Therefore, the moisture-removal process was primarily carried out by keeping the moisture-absorbing molecular beads around the NF, and the solution was kept in a shaking incubator at 120 RPM and a temperature of 60 °C up to 12 h. Secondly, the NF was vacuum treated at an ambient temperature, and the pressure was kept high using a vacuum oven for further moisture removal. The moisture content was analyzed using the Karl Fischer titration (KFT) method.

### 2.3. Measurement Error of Instrument

The expression for combined uncertainty error for ACBDV and thermal conductivity is given in (1):

(1) $$U_{c}=\sqrt{U_{1}{}^{2}+U_{2}{}^{2}+U_{3}{}^{2}}$$

where *U*_1_ is the standard uncertainty of the instrument, *U*_2_ is the standard uncertainty for gap gauge/probe, and *U*_3_ is the standard uncertainty of the electrode dimension/measurement. The measurement uncertainty of ACBDV and thermal conductivity is shown in Table 3 and Table 4, respectively.

## 3. Results and Discussion

The thermal conductivity and ACBDV studies were performed to understand the cooling and insulation characteristics of the developed NF. The detailed mechanism of the enhancement was studied and compared theoretically.

### 3.1. Stability Analysis by Zeta Potential

The stability of the NFs was carried out for different concentrations, as shown in Figure 1b. Three NPs such as h-BN, Eh-BN, and TiO_2_ were dispersed in MO at three different concentrations: 0.01, 0.05, and 0.1 wt.%. The constancy of the prepared solution was interpreted with the measured value of zeta potential. The variation of the NP concentration in the supernatant of NF was measured using the absorbance of UV-vis spectrophotometer with sedimentation time. In the NF, the supernatant NP concentration directly influenced the absorbance of the suspended particles. The value of zeta potential more than 40 mV was observed to be more stable [22]. It was detected from Figure 1 that the constancy of the NFs declined speedily with an upsurge of concentration of the NPs. The Eh-BN/MO-NF with a concentration of 0.01 wt.% of NPs was seen to be stable up to four days. In this paper, the role of zeta potential in the various other parameters such as viscosity, electrophoretic mobility, and dielectric constant is further studied in Section 4. Moreover, the effect of zeta potential on the dielectric breakdown of the NFs is also investigated.

### 3.2. Thermal Conductivity

The cooling performance of the developed NFs was studied by monitoring the thermal conductivity of all the oil samples and comparing them with MO. The thermal conductivity was measured with the help of the transient hot-wire method (THM) using the KD2 Pro thermal properties analyzer. A certain extent of the probe is dipped into the fluid and excited electrically to transmit the heat. The alteration in the resistance due to the change in temperature is estimated as a function of time. The slope of heating power and the alteration in temperature with a log time scale is the measure of thermal conductivity. THM evaluates the possible effect of temperature, time, NP concentration, and NP size on thermal conductivity.

Theoretically, the thermal conductivity of the NFs is achieved by using the Feng–Kleinstreuer (F-K) analytical expression, as given in Equation (1) [23]. The addition of minute nanofiller concentration results in an appreciable increment in thermal conductivity because of the large surface area of the NPs. At a greater nanofiller concentration, the enhancement of thermal conductivity drops, violating Maxwell’s classical theory,

(2) $$k_{eff}=\left(1+\frac{3\left(\frac{k_{p}}{k_{bf}}-1\right)\phi }{\left(\frac{k_{p}}{k_{bf}}+2\right)-\left(\frac{k_{p}}{k_{bf}}-1\right)\phi }\right)k_{nf}+\frac{8250k_{B}}{\pi \mu_{bf}d_{p}}{\left(\rho c_{p}\right)}_{nf}\phi^{2}\bar{T}\ln\bar{T}-\bar{T}$$

where *k_p_*, *k_bf_*, and *k_nf_* are the thermal conductivities of the particles, base fluid, and NF, respectively. *φ*, *µ_bf_*, *c_p_* and *ρ* are the volume fraction of dispersed NPs, coefficient of viscosity of the base fluid, specific heat, and density of the fluid, respectively. The diameter of the NP and the average temperature of the NF are *d_p_* and $\bar{T}$, respectively.

The heat-transfer characteristic of the NFs and MO at elevated temperatures were studied for stable concentration of 0.01 wt.% of NPs. NFs were heated to six different stages of temperature from 25 to 50 °C, and the thermal conductivity was measured using THM. With the help of the attached temperature sensor, the oil sample’s instantaneous temperature was detected.

**Figure 2:**
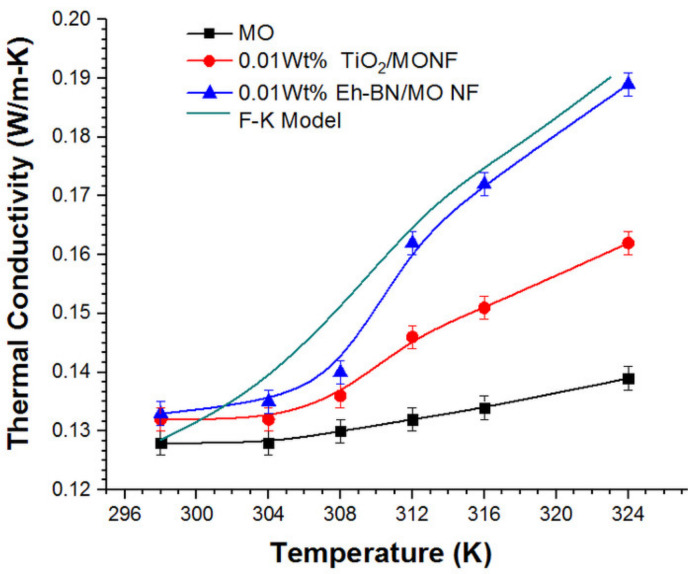
Thermal conductivity of NFs with the rise in temperature.

The thermal conductivity of MO and 0.01 wt.% NFs at different temperatures were studied and compared analytically with the F-K analytical model, as shown in Figure 2. There is a considerable augmentation in thermal conductivity of 0.01 wt.% Eh-BN/MO-NF compared to MO and TiO_2_/MO-NF. Owing to the Brownian motion of the NPs, there is an enhancement in the thermal conductivity. At the interface of the particle and the liquid, there is liquid layering, which also accounts for the higher heat transfer. The high aspect ratio of the Eh-BN nanofillers is also responsible for better heat conduction. Hence, a significant enhancement in cooling performance was observed for Eh-BN/MO-NF [8]. Owing to the high aspect ratio of the NPs, surface charges are enhanced, and hence the thermal conductivity of the NFs improves. It is seen from Figure 2 that the exfoliation of bulk h-BN powder to 2D nanosheets enhanced the thermal conductivity of the Eh-BN/MO-NF from 4 to 36% with the upsurge in temperature, while for TiO_2_/MO-NF, the enhancement in thermal conductivities was 3 to 16%. As Eh-BN based NF has the potential for heat-transfer capacity, it is likely to be the better coolant than MO and TiO_2_-based NF.

### 3.3. AC Breakdown Voltage (ACBDV)

**Figure 3:**
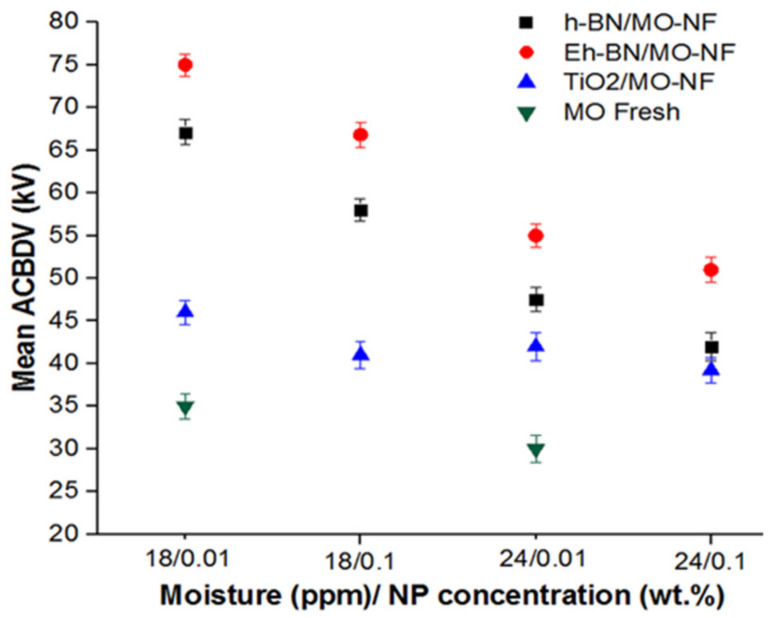
Mean ACBDV of MO and NFs at 18 and 24 ppm moisture content.

To measure the electric stress and withstand capacity of the dielectric fluids, a measurement of ACBDV was carried out. The analysis of ACBDV was carried out using the BAUR DTA 100 C oil tester, following ASTM D1816. The facility is availed of the Insulating Oil Testing Laboratory (IOTL), PGCIL, Nagaon, Assam, India. In this measurement, the VDE electrodes used were maintained at a distance of 2.5 mm from each other. The applied steady voltage was 2 kV/s at a line frequency of 50 Hz at room temperature. A short holding time of 10 min was allotted to the dielectric fluid before applying the gradual voltage. In between two successive measurements for a certain liquid, a holding time of 1 min was provided. The five consecutive ACBDV values were obtained for one batch of fluid, and the mean ACBDV of fresh MO and three different batches of NFs for moisture content of 18 and 24 ppm are presented in Figure 3.

It was observed from the figure that at lower moisture content, the ACBDV value was found to be superior compared to higher-moisture-content samples. Since moisture is polar in nature, its contamination in the fluid during the applied field releases free electrons owing to the collision of available electrons in the fluid. This enhances the streamer propagation in the fluid and makes early breakdown, and hence ACBDV decreases. The enhancement in ACBDV of TiO_2_/MO-NF and Eh-BN/MO-NF compared to MO was 31.4, 26.7% and 114.2, 85% at 18 and 24 ppm, respectively.

**Figure 4:**
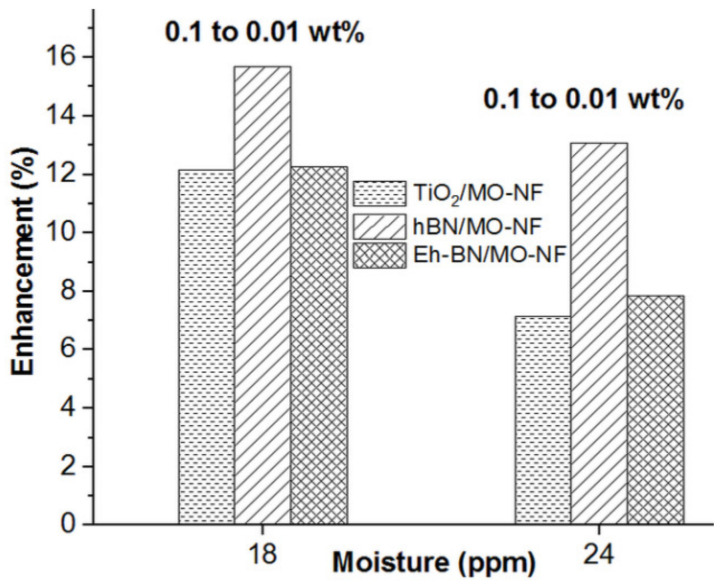
Enhancement of ACBDV of NFs between 0.01 and 0.1 wt.% of NP concentration for a moisture level of 18 and 24 ppm.

The enhancement of ACBDV for three different batches of NFs with TiO_2_, Eh-BN, and h-BN as NPs at 18 and 24 ppm is reported in Figure 4. The comparative percentage enhancement between 0.01 and 0.1 wt.% of NP at 18 and 24 ppm was studied. It was detected that if the moisture percentage rises, the subsequent ACBDV value decreases for all the batches of NFs. It was observed from the comparative analysis that with the rise in NP concentration in the MO, the ACBDV value decreases. The colloidal solution of higher NP concentration weakened in electric double-layer repulsion and formed clogging among the dispersed particles. As a result, the mean ACBDV value fell for 0.1 wt.% compared to 0.01 wt.% of nanofiller dispersion, even at distinct moisture levels.

**Figure 5:**
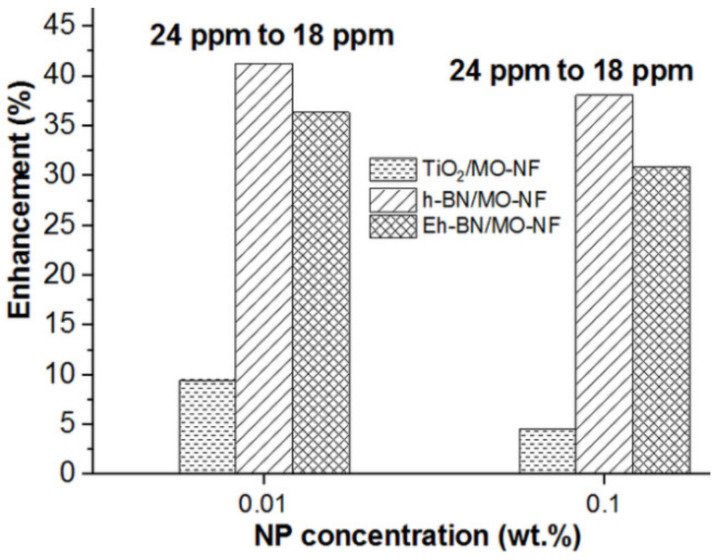
Enhancement of ACBDV of NFs between 18 and 24 ppm moisture level for 0.01 and 0.1 wt % of NP concentration.

The enhancement in ACBDV between 18 to 24 ppm is shown in Figure 5. It is observed that the hydrophilic nature of TiO_2_ NP helps to hold the moisture molecule, whereas moisture content minimally affects the Eh-BN-based NF. Therefore, the value of ACBDV for Eh-BN/MO-NF is superior to TiO_2_/MO-NF. The detailed analysis of the insulation failure for different batches of NFs and MO was studied using statistical analysis of the ACBDV.

**Figure 6:**
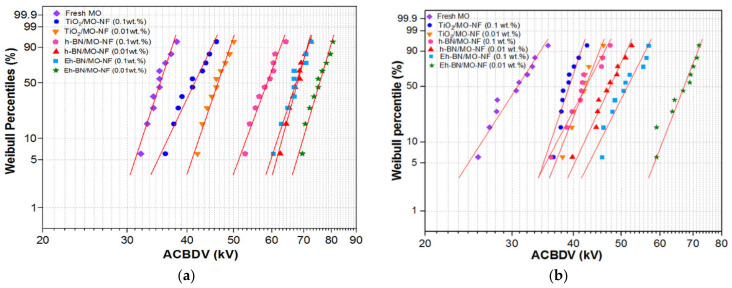
Weibull probability distribution fluid samples (**a**) 18 ppm (**b**) 24 ppm.

The Weibull [10,11] two-parameter model was estimated for the statistical analysis of the ACBDV data for TiO_2_, h-BN, and Eh-BN-based NFs and the conventional MO. The ACBDV tests were carried out at 0.1 and 0.01 wt.% NP concentration and moisture levels of 18 and 24 ppm. The probability of failure is given in Figure 6a,b. The Weibull distribution parameters are presented in Table 5.

The cumulative density function (*F*(*x*)) for the two-parameter model is expressed in Equation (2) and is plotted with a confidence interval of 95%, and is as below,

(3) $$F(x:\alpha ,\beta )=1-\exp{\left(-(x/\alpha )\right)}^{\beta };x>0$$

where *x* is the ACBDV, *α* is the scale parameter, and *β* is the shape parameter.

The failure probabilities of BDV at 50 and 63.2% at moisture levels of 18 and 24 ppm are given in Table 6. The improvement in the ACBDV for Eh-BN/MO-NF at 0.01 wt.% is highest at both 63.2% and 50% failure probabilities compared to other oil samples. The failure probability at 63.2% is very crucial, as it represents the characteristic life of the sample. The charge-dynamics study was carried out to understand the physics of BDV of the NFs, and is explained below.

## 4. Charging Dynamics

The NPs, when dispersed in the insulating fluid, capture the free charge due to polarization or ionization when electrical stress is applied. This makes it difficult for the carriers to move in between the electrodes. This, in turn, increases the breakdown strength of the dielectric in between the electrodes as a higher amount of electrical stress is required to form a conducting channel or streamer for the breakdown. With the increase in the concentration of the NPs in the base fluid, the enhancement in BDV is more pronounced. However, after a certain threshold, the BDV starts to decline. This phenomenon happens because of the overlapping of the electrical double layer (EDL) formation on the surface of the nanoparticle on the application of electrical stress [24]. This overlapping creates a highly conductive region, inducing the charge carriers to propagate and form a conducting channel causing an earlier breakdown.

**Figure 7:**
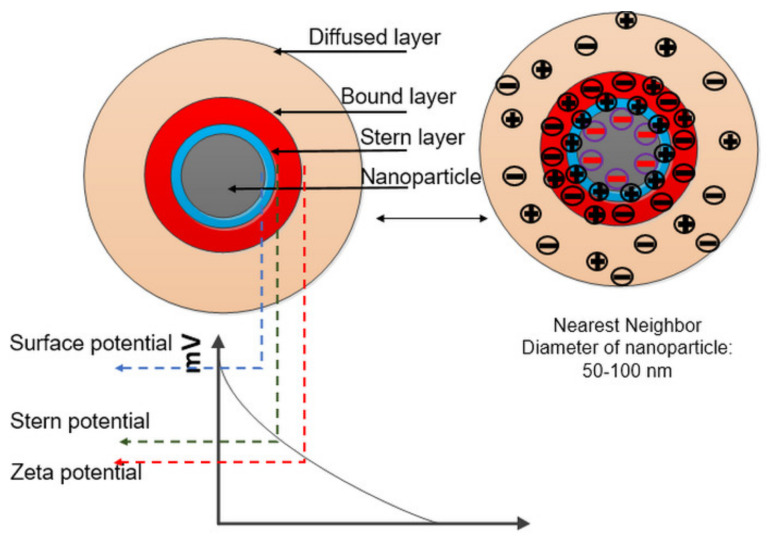
Study of zeta potential with the surface layer of the NPs.

This liquid layer surrounding the particle forms three regions, as shown in Figure 7. The first is known as the surface-charge region. When the particle is negatively charged, it will attract cations in the second layer, known as the stern or compact layer, where the attractive forces are the highest. The third layer, known as the diffuse region, has the ions of both charges. In this layer, a higher number of anions will be attracted by the stern layer. There is a boundary within the diffuse layer, and this is known as the slip plane or hydrodynamic shear plane. At this plane, the particle’s zeta potential is estimated. The particle travels owing to the Brownian motion, so along with it, the ions within the slip plane also travel because they are strongly attracted to the particle, but ions outside of the plane do not travel.

Nanofluid is a colloid system, where the collision between the particles occurs due to Brownian motion and an external electric field. Due to this collision, the particles will either aggregate or separate depending on the attractive Van der Waals potential and repulsive electrostatic potential. Depending on which potential is higher, the NPs will eventually stay dispersed or agglomerated. The collision leads to a lessening of the distance between the NPs, and the attractive Van der Waals force causes agglomeration. A repulsive electrostatic potential results from the interaction between the EDLs surrounding the particles. When concentration increases, agglomeration occurs, the value of the zeta potential decreases further and stability is reduced, and the NPs tend to settle down, causing the process of electron scavenging to slow down and BDV reduces.

When high-voltage stress is applied, electrohydrodynamic behavior of the Eh-BN and TiO_2_-based NFs leads to the increased breakdown strength owing to the electrophoresis effect [25]. The presence of NPs in the base fluid leads to the formation of a potential well on the surface of the NP, so the field lines converge on the NPs and electron scavenging occurs, which prevents the electrons from moving towards the anode and slower down the streamer formation, which eventually enhances the breakdown voltage. During the electron-capturing process, the stray electrons generated on the application of electrical stress are deposited on the surface of the NPs. However, after a certain threshold of surface deposition, no more electrons are adsorbed on the surface, and with increased concentration of NPs in the oil, the proximity of NPs leads to the formation of conducting channels for the streamer to propagate and hence the breakdown strength starts decreasing.

The electrohydrodynamics effect is caused by the electrophoretic force, which leads to a decrease in the viscosity of the NF. Studies show that an external electric field contributes to the movement of the NPs in the insulating fluid. This velocity of the NPs is attributed to Brownian motion and the electrophoretic force. Due to the longer wavelength of the electrophoretic force, it is able to dislocate NPs to longer distances and hence modifies the viscous behavior of the liquid as the velocity of the NPs increases. This force is treated as a secondary motion because it is a result of the external electrical field. In absence of the field, only Brownian motion is prevalent, and hence displacement of NPs is negligible [26].

The viscous fluids hinder electrophoretic mobility, which is proportional to zeta potential. The velocity of a particle, which is also known as the electrophoretic mobility, is related to Zeta potential and viscosity by the Smoluchowski approximation given below, also called Henry’s Equation (4) [27]:

(4) $$\mu =\frac{2\varepsilon_{r}\varepsilon_{o}\zeta f(\kappa a)}{3\eta }$$

where *μ* is the electrophoretic mobility, *ε* is the dielectric constant, *ζ* is the zeta potential, *f*(*κa*) is called the Henry’s function, and *η* is the viscosity.

The variable *κ*, called the Debye length, is the reciprocal length and *κ*−1 is a measure of the “thickness” of the EDL. The factor ‘a’ denotes the radius of the particle and therefore ‘*κa*’ gives the ratio of the particle radius to electrical double-layer thickness.

Considering the Huckel approximation for small particles (<<200 nm), the Henry function is considered as unity, then the modified equation becomes Equation (5):

(5) $$\mu =\frac{2\varepsilon_{r}\varepsilon_{o}\zeta }{3\eta }$$

From the above equation, it is observed that for higher zeta potential, the electrophoretic mobility is increased. This, in turn, reduces the viscosity, which is desirable and aids in achieving superior stability of the NPs in the base fluid. The stable suspensions eventually help in obtaining a higher breakdown strength.

**Figure 8:**
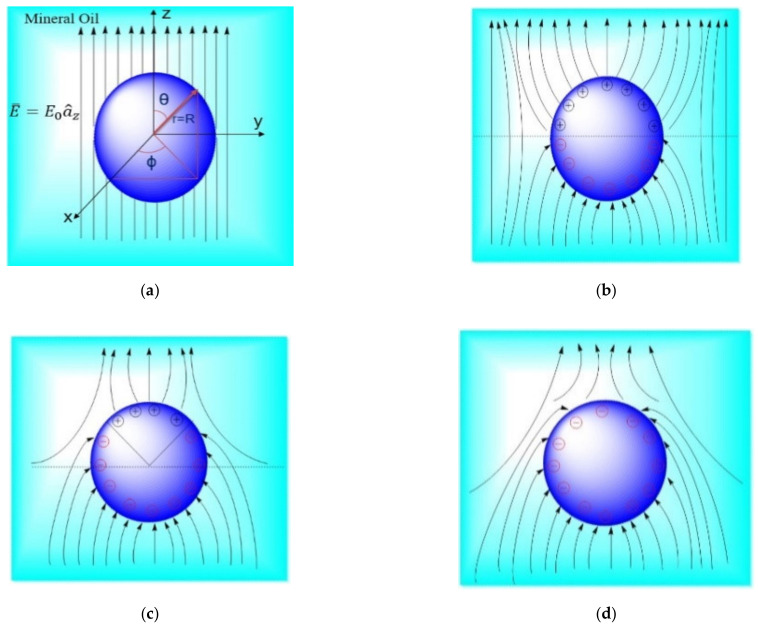
Charging of the NP (**a**) particle exposed to an external field, (**b**) ionization or polarization of the NP, (**c**) depletion of the positive ions, and (**d**) complete depletion of the positive ions.

In order to understand the aforementioned augmentation in the electrical breakdown strength of the NFs, two cases with two different NPs are considered. An Eh-BN dielectric with an average radius R of 75 nm, permittivity *ε*_2_ = 4*ε*_0_ F/m, and conductivity *σ*_2_ = 10^−13^ is considered; and a TiO_2_ semiconducting NP with average radius *R* of 12.5 nm, permittivity *ε*_2_ = 31*ε*_0_ F/m, and conductivity *σ*_2_ = 10^−6^ S/m is considered. The base fluid surrounding the NPs is MO with permittivity *ε_1_*(=2.03*ε*_o_ F/m) and conductivity *σ*_1_ (=10^−12^ S/m) is shown in Figure 8a.

The time-varying component of the radial electric field in the MO outside the NP due to the excitation, *E*_o_ is [2]:

(6) Er→(r≥R+,θ)=a^rE01+KCεe−tτr+KCσ(1−e−tτr)cosθ

where $K=\frac{2R^{3}}{r^{3}}$, $C_{\sigma }=\frac{\sigma_{2}-\sigma_{1}}{2\sigma_{1}+\sigma_{2}}$ and, $\tau_{r}=\frac{2\varepsilon_{1}+\varepsilon_{2}}{2\sigma_{1}+\sigma_{2}}$ is the relaxation time.

Since the NP has finite conductivity; it takes a finite time to place the charges on the surface of the NPs depending on the particle and oil conductivities and permittivities. It is observed from Figure 8b that when the NP is exposed to an external electric field, the positive and negative ions are generated in the upper half (0 < *θ* ≤ π/2) and lower half (π/2 < *θ* ≤ π) of the particles without any space charge in the bulk. As time progress, the electrons originated from ionization or polarization deposit on the particle and curtail the positive ions, so positive ions are confined in *0* < *θ* < *θ_c* and the negative ions in *θ*_*c* < *θ* < π, as shown in Figure 8c. In this situation, the modified expression of the electric field on the surface of the NP dispersed in MO in the presence of deposited surface charge *Q(t)* is Equation (7) [2,28]:

(7) Er(r≥R,θ)=E01+KCεe−tτr+KCσ(1−e−tτr)cosθ+Q(t)4πε1r2

As the deposition of the electrons on the nanoparticle continues, the positive charge diminishes to zero (i.e., *E_r* (*r* = *R,θ*) ≥ 0) and the particle is totally charged by a saturation charge *Q_s_*, as shown in Figure 8d. The saturation charge *Q_s_* for the NP derived from *E_r* (*r* = *R*,*θ*) = 0 is Equation (8):

(8) $$Q_{s}=-12\pi \varepsilon_{1}R^{2}E_{o}\frac{\varepsilon_{2}}{2\varepsilon_{1}+\varepsilon_{2}}$$

The charging rate of the NP derived from Equation (6) is given as presented in Equation (9):

(9) $$\frac{dQ(t)}{dt}=\frac{3Q_{s}}{\tau_{pc}C(t)}{\left[\frac{Q(t)}{Q_{s}/\left(\frac{\varepsilon_{2}}{2\varepsilon_{1}+\varepsilon_{2}}\right)}-\frac{C(t)}{3}\right]}^{2}$$

where the charging time constant of the NP is expressed in Equation (9):

(10) $$\tau_{pc}=\frac{4\varepsilon_{1}}{\left|-\rho_{e}\right|\mu_{e}}$$

and

(11) C(t)=1+2Cεe−tτr+2Cσ(1−e−tτr)

**Figure 9:**
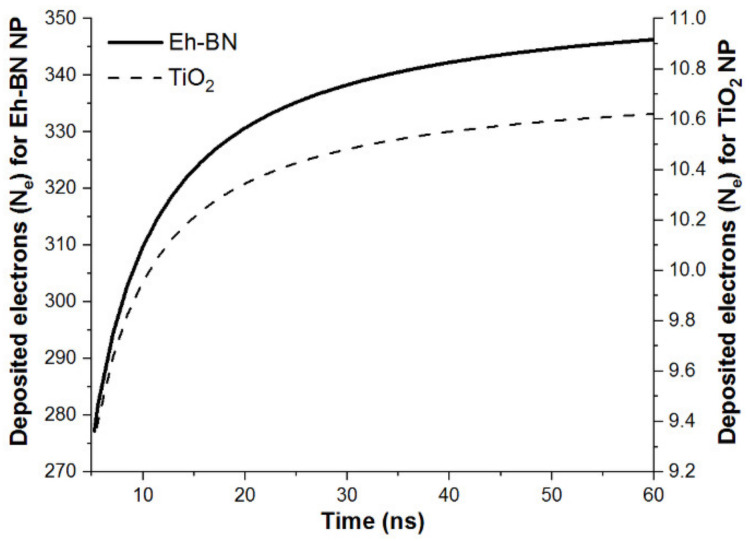
Charging characteristics of Eh-BN and TiO_2_ in MO.

Solving Equation (8), using MATHEMATICA, the dynamic-charge accumulation *Q(t)* on the Eh-BN and TiO_2_ NP are shown in Figure 9.

The number of electrons absorbed on the surface of the Eh-BN and TiO_2_ NP is 354 and 11, respectively, as shown in Figure 9 and Table 7. The total number of particles in 0.01 wt.% of Eh-BN and TiO_2_ are calculated as 2.43 × 10^20^ and 7.54 × 10^19^, respectively. This decelerates the ionization process in Eh-BN/MO-NF, and hence BDV increases. From Table 7, it is noted that the captured saturation electrons for the Eh-BN/MO-NF are higher than that of TiO_2_-based NF. Since Eh-BN NPs capture a higher number of electrons in the NF, the ionization process delays, and hence the BDV increases compared to TiO_2_-based NF and MO.

## 5. Conclusions

In this research work, thermal conductivity and electrohydrodynamics analysis were carried out for different batches of NFs. Two types of NPs, h-BN and TiO_2_, were considered to prepare the NFs. Bulk h-BN NP of size 1μm was exfoliated into 2D nanosheets to enhance the surface area of exfoliated h-BN (Eh-BN). The thermal conductivity and ACBDV studies of the prepared NF were performed and the charging-dynamics study verified the enhancement in ACBDV of the Eh-BN NF. The important conclusions of this work are as follows:

- The dispersion of 0.01 wt.% of Eh-BN NP is observed to be stable for a longer period.
- The high aspect ratio and greater surface charges in Eh-BN NP are largely responsible for higher thermal conductivity in Eh-BN/MO NF.
- Higher moisture contamination in the MO and NFs reduces the ACBDV. However, Eh-BN NP has a minimal affinity towards moisture and augments the ACBDV compared to MO and other batches of NFs.
- From the charge-dynamics analysis, it is observed that the Eh-BN NP scavenges a higher number of free electrons compared to other NPs, and hence the initiation of the streamer is arrested, which leads to enhancement in the ACBDV for Eh-BN/MO-NF compared to MO and other NFs.

Considering the aforementioned advantages of the Eh-BN NF over existing MO, it is likely to improve the cooling and insulation characteristics of the dielectric fluid.

## Figures and Tables

**Table 1:** Specifications of MO.

Characteristic	Specification
Formula	C_n_H_2n+2_
Density (gm/cc)	0.828
Kinematic viscosity at (cSt)	0.0123
Interfacial tension (IFT)at (N/m)	0.047
Flash point in (°C)	146
Pour point in (°C)	−18
Tan delta at 90 °C (Max).	0.0085
Water content (ppm)	25
AC breakdown voltage (kV)	30
Thermal conductivity (W/m-K)	0.128

**Table 2:** Specifications of NP.

Characteristic	Specification	
	TiO_2_	Eh-BN
Purity (%)	99.5	98
Size (nm)	21	0.1
Density (g/cm^3^)	3.9	2.29
Dielectric constant	31	3–4
Thermal conductivity (W/m-K)	11	300
Electrical resistivity (Ω-cm)	10^14^	10^15^
Thermal expansion coefficient (°C)	11.5 × 10^−6^	4 × 10^−6^

**Table 3:** Measurement uncertainty for ACBDV.

Source of Uncertainty	% Error (±)
Instrument accuracy (*U*_1_)	0.916
Gap gauge (*U*_2_)	0.44
Electrode (*U*_3_)	0.168
Combined uncertainty (*U_c_*)	1.03

**Table 4:** Measurement uncertainty for thermal conductivity.

Source of Uncertainty	% Error (±)
Instrument accuracy (*U*_1_)	0.5
Electrical probe (*U*_2_)	0.2
Measurement (*U*_3_)	0.1
Combined uncertainty (*U_c_*)	0.547

**Table 5:** Weibull distribution parameters.

Oil Samples							
18 ppm							
	MO	TiO_2_ 0.01	TiO_2_ 0.1	h-BN 0.01	h-BN 0.1	Eh-BN 0.01	Eh-BN 0.1
α	35.7	47.1	42.4	68.8	59.8	76.6	68.5
β	21.5	20.8	14.8	25.8	19	23.38	21.5
24 ppm							
α	31.7	43.1	39.8	48.5	43.5	68.4	52.8
β	11.3	18.4	21.6	15.6	13.8	18.7	14.4

**Table 6:** Failure probabilities at 18 ppm and 24 ppm.

Moisture Level	Oil Samples	63.2%		50%	
		KV	% Rise	kV	% Rise
18	MO	35	118.5	35	114.2
	TiO_2_-0.01	47	62.7	46	63
	TiO_2_-0.1	43	77.9	41	82.9
	h-BN-0.01	68.6	11.5	68	10.3
	h-BN-0.1	60.5	16.4	58.5	28.2
	Eh-BN-0.01	76.5	0	75	0
	Eh-BN-0.1	66.8	14.5	66.8	12.2
24	MO	32.2	114.9	30.8	119.1
	TiO_2_-0.01	42.1	64.3	42	60.7
	TiO_2_-0.1	39.2	76.5	38.5	75.3
	h-BN-0.01	49	41.2	47	43.6
	h-BN-0.1	42	64.7	41.5	62.6
	Eh-BN-0.01	69.2	0	67.5	0
	Eh-BN-0.1	52	33	50.5	33.6

**Table 7:** Electron captures by the NPs.

NPs	*Q_s_* per NP × 10^−18^ (C)	*N_e_* per NP	*N_np_* in 100 gm of MO × 10^19^	Total *N_e_* × 10^19^
TiO_2_/MO	−1.72	11	7.54	83
Eh-BN/MO	−5.675	354	2.43	860.22

Note: *N_e_* and *N_np_* are the numbers of electrons and the total number of NPs.
